# Linguistic and Musical Syntax Processing in Autistic and Non‐Autistic Individuals: An Event‐Related Potential (ERP) Study

**DOI:** 10.1002/aur.70038

**Published:** 2025-04-05

**Authors:** Jiayin Li, Anna Petrova, Zivile Bernotaite, Maleeha Sujawal, Chen Zhao, Hiba Ahmed, Cunmei Jiang, Fang Liu

**Affiliations:** ^1^ School of Psychology and Clinical Language Sciences University of Reading Reading UK; ^2^ Department of Psychology, Royal Holloway University of London London UK; ^3^ School of Health Sciences University of Manchester Manchester UK; ^4^ Music College Shanghai Normal University Shanghai China

**Keywords:** autism, language, music, P600, syntax

## Abstract

Syntactic processing in both language and music involves combining elements—such as words or chords—into coherent structures. The Shared Syntactic Integration Resource Hypothesis (SSIRH) was introduced based on observations of similar neural responses to syntactic violations across both domains. This hypothesis suggests that difficulties in syntactic processing in one domain may result in similar challenges in the other. The current study tested the SSIRH in autism, a neurodevelopmental condition often associated with language difficulties but relatively preserved musical abilities. Thirty‐one autistic and 31 non‐autistic participants judged the acceptability of syntactically congruent and incongruent sentences and musical sequences while their neural responses were recorded using electroencephalography. Autistic participants exhibited a reduced and delayed P600 effect—a marker of syntactic integration—across both domains, despite achieving similar behavioral accuracy to the non‐autistic group. These findings suggest parallel difficulties in syntactic processing in autism for both language and music, providing support for the SSIRH. This is the first study to directly examine real‐time syntactic integration in both domains in autistic individuals, offering novel insights into cross‐domain syntactic processing in autism and contributing to a deeper understanding of language and music processing more broadly.

**Summary**

- Our study looks at how autistic and non‐autistic adults process patterns in language and music.
- These patterns, called syntax, are the rules we use to build sentences or melodies.
- We tested how people respond when these rules are broken, using tasks that measured their accuracy and brain activity.
- Autistic adults were just as accurate as non‐autistic adults, but their brain responses were slower and less active.
- This suggests that their brains process patterns differently.
- These findings could help us better understand autism and develop ways to support learning in language and music.

## 1 Introduction

Language and music are complex cognitive systems that, while distinct, exhibit significant structural similarities. Both systems consist of perceptually distinct elements organized into hierarchically structured sequences governed by syntactic principles (Deutsch 1999; Lerdahl 2005). These principles determine how elements—such as words in language and tones in music—combine to form meaningful sequences (Koelsch et al. 2005; Patel 2003). These similarities raise the possibility that the processing of syntactic structures in music may share commonalities with linguistic syntax.

Theoretical backing for this idea is provided by the Shared Syntactic Integration Resource Hypothesis (SSIRH; Patel 2003, 2010). Linguistic syntax depends on lexical knowledge and hierarchical combinatorial rules, whereas musical syntax is structured around tonal hierarchies and rhythmic organization processed using domain‐specific representations (Peretz and Coltheart 2003). While recognizing these differences, the SSIRH suggests that both domains draw on shared resources to integrate elements into structured sequences. Supporting evidence comes from neurophysiological studies showing similar brain responses for both types of processing (Koelsch et al. 2000; Maess et al. 2001) and neuroimaging studies identifying overlapping brain regions involved in syntactic processing for language and music (Janata et al. 2002; Koelsch et al. 2002; Kunert et al. 2015). In contrast, Peretz et al. (2015) propose that while the processing of language and music shares some overlapping brain regions, they engage domain‐specific representational networks. Therefore, further research is needed to explore this ongoing debate.

Populations with syntactic impairments offer a window into the SSIRH. Patel (2013) proposed that individuals with compromised syntactic integration networks should exhibit parallel deficits in both language and music. This has been explored in congenital amusia, agrammatic aphasia, and children with developmental language disorder (DLD) (Chiappetta et al. 2022; Jentschke et al. 2008; Slevc et al. 2016; Sun et al. 2018), but not yet in autism. Autism spectrum disorder (ASD) is characterized by challenges in social communication, stereotyped behaviors, and restricted interests (American Psychiatric Association 2013). Interestingly, while autistic individuals often exhibit difficulties in language and socio‐communicative abilities, they frequently show preserved or even enhanced musical abilities (O'Connor 2012; Ouimet et al. 2012). While research on this language‐music dissociation has largely focused on pitch or pitch‐related acoustic processing (Jiang et al. 2015; Lai et al. 2012; Sharda et al. 2015; Wang, Ong, et al. 2023; Wang, Schoot, et al. 2023), higher‐level syntactic processing remains understudied in autism (DePriest et al. 2017; Zhao et al. 2024).

In the language domain, autistic individuals face significant challenges with syntactic processing, particularly when dealing with complex sentence structures requiring greater computational resources. They often struggle with decoding relative clauses and managing long‐distance dependencies (Durrleman et al. 2015; Riches et al. 2010), but perform well on simpler tasks, such as identifying indirect objects with explicit prepositions (Stockbridge et al. 2014). Studies reveal that autistic individuals with DLD encounter difficulties in distinguishing and interpreting pronouns (Perovic et al. 2013; Terzi et al. 2012) and in repeating sentences with complex morphosyntactic structures (Manenti et al. 2024; Riches et al. 2010). Despite comparable accuracy to non‐autistic peers with DLD, autistic individuals show distinct error patterns and processing strategies, suggesting that language abilities alone do not fully explain their atypical syntactic processing (Riches et al. 2010; Sukenik and Friedmann 2018). Additionally, these syntactic difficulties persist even among autistic individuals with typical nonverbal IQs (Perovic et al. 2013; Riches et al. 2010; Terzi et al. 2012; Zebib et al. 2013), highlighting the need for further research into the mechanisms behind these challenges.

Conversely, the limited research on musical syntax in autism suggests intact processing abilities. Heaton et al. (2007) found no significant differences between autistic and non‐autistic individuals in judging the coherence of harmonic sequences. Similarly, Quintin et al. (2013) observed that autistic adolescents performed comparably to non‐autistic peers in a task that required participants to arrange musical segments into structurally well‐formed sequences, ensuring coherence in harmonic progression. Zhao et al. (2024) reported intact musical prediction across production and perception tasks for both Mandarin‐ and English‐speaking autistic individuals. These findings suggest that while autistic individuals may struggle with linguistic syntactic integration, their ability to process musical syntax appears unaffected.

According to the SSIRH, if syntactic integration relies on cross‐domain computations, autistic individuals' difficulty in processing hierarchical structures in language should also extend to music. However, as reviewed above, previous behavioral evidence does not support this. This discrepancy may arise since no studies have directly compared linguistic and musical syntactic processing using matched tasks, leaving open the question of whether intact processing of musical syntax can coexist with impaired processing of linguistic syntax in autism. Additionally, methodological inconsistencies across the aforementioned studies on linguistic syntax, along with limited real‐time processing data, may contribute to the ambiguity. Variations in task designs and the focus on specific aspects of syntax make it difficult to determine whether autistic individuals experience general syntactic difficulties across all structures or pattern‐specific difficulties, such as dependencies (e.g., relative clauses, long‐distance dependencies; Durrleman et al. 2015; Riches et al. 2010), referential processing (e.g., pronouns; Perovic et al. 2013; Terzi et al. 2012), and morphosyntactic complexity. While resolving these distinctions is beyond the scope of the current study, we address this concern by directly comparing syntactic integration in both language and music domains through matched tasks adapted from Patel et al. (1998).

Patel et al. (1998) examined online syntactic processing using the P600 response, an ERP component observed around 600 ms after stimulus presentation. The P600 reflects the timing and neural resources involved in syntactic integration, originally linked to syntactic violations (Friederici et al. 1998) and later associated with broader integration challenges (Kaan et al. 2000). Patel et al.'s (1998) study used harmony‐based chord sequences and syntactically structured sentences, engaging similar syntactic processes. Musical sequences included target chords varying by key (in‐key, nearby key, or distant key), while sentences featured target words that were easy, difficult, or impossible to integrate into the preceding structure. The study found that out‐of‐key chords and syntactically incongruent words elicited indistinguishable P600 responses, indicating shared neural mechanisms for syntactic integration across music and language. A replication study by de Leeuw et al. (2019) reinforced these findings. Using Patel et al.'s (1998) paradigm with minor modifications, they observed comparable P600 effects for both harmonic irregularities and syntactic violations, with only slight differences in spatial distribution.

Building on this paradigm, we examined whether autistic adults with intact language and cognitive abilities show similar neural responses to linguistic syntax and musical harmony, using the P600 as a marker of syntactic integration. Following the SSIRH, we hypothesized that if autistic individuals experienced cross‐domain syntactic integration difficulties, they would exhibit comparable reductions in P600 responses to syntactic violations in both language and music. However, given previous behavioral findings suggesting intact musical but impaired linguistic syntax processing in autism (e.g., Heaton et al. 2007; Zhao et al. 2024), it is also possible that autistic individuals might show a typical P600 response to musical violations while exhibiting a reduced P600 response in the language task.

## 2 Methods

### 2.1 Participants

Thirty‐one autistic adults and 31 non‐autistic adults aged 18–42 participated in the experiment. Participants were recruited through within‐school outreach, social media, and engagement with local organizations. All participants were right‐handed native English speakers with normal or corrected‐to‐normal vision and passed a hearing screening using an Amplivox manual audiometer, demonstrating normal hearing in both ears at 25 dB for frequencies of 0.5, 1, 2, and 4 kHz. Both groups had no current speech, language, or communication needs, and participants with such impairments were excluded from the study. The autistic group had confirmed diagnoses from professional clinicians, supported by clinical reports. None of the non‐autistic participants had a family history of ASD or had been diagnosed with ASD, which was further confirmed by their Autism Spectrum Quotient (AQ; Baron‐Cohen et al. 2001) scores.

To account for potential cognitive influences, we collected a comprehensive set of background measures including nonverbal IQ using the Raven's Standard Progressive Matrices Test (Raven et al. 1998), receptive vocabulary skills using the Receptive One‐Word Picture Vocabulary Test, Fourth Edition (ROWPVT‐4) (Martin and Brownell 2011), and verbal short‐term memory using the digit span task, implemented on the Psychology Experiment Building Language Test Battery software (Mueller and Piper 2014). Given the importance of musical experience and ability for our study, we collected data on musical training, including years of formal training across all instruments, through a questionnaire (Pfordresher and Halpern 2013). Musical perception ability was evaluated using the three pitch‐based subtests (i.e., scale, contour, and interval) from the Montreal Battery of Evaluation of Amusia (MBEA; Peretz et al. 2003). In each subtest, participants were presented with pairs of melodies, and they were asked to identify whether each pair was the same or different. Participants were excluded if their Raven's IQ scores fell at or below the fifth percentile for their age group or if they scored 70 or below on the standard score measure of ROWPVT‐4. In total, five participants (four autistic and one non‐autistic) were excluded for not meeting these criteria. Demographic information and statistical comparisons were summarized in Table 1. Welch's two‐sample *t*‐tests showed no significant group differences in chronological age, receptive vocabulary, nonverbal reasoning ability, verbal short‐term memory, musical perception ability, or musical training background. However, the autistic group scored significantly higher on the AQ, indicating higher autistic traits.

**TABLE 1:** Characteristics of the autistic (*n* = 31) and non‐autistic groups (*n* = 31).

Variables	Autistic	Non‐autistic	*W*	*p*	Rank‐biserial correlation
	Mean (SD)	Mean (SD)			
Gender (female:male:others)	18:11:2	26:5:0			
Age	25.32 (6.37)	24.90 (6.71)	515.0	0.63	0.07
Musical training	4.21 (5.57)	5.14 (6.35)	439.5	0.55	0.09
Nonverbal reasoning (RSPM raw core/60)	54.19 (3.47)	53.87 (3.97)	491.5	0.88	0.02
Nonverbal reasoning (RSPM standard core)	49.36 (24.69)	49.52 (29.76)	498.0	0.80	0.04
Receptive vocabulary (ROWPVT‐4 raw score/190)	167.45 (10.34)	168.84 (8.49)	472.5	0.92	0.02
Receptive vocabulary (ROWPVT‐4 standard score)	109.48 (15.16)	104.75 (11.39)	449.0	0.66	0.07
MBEA scale (/30)	26.03 (2.33)	26.39 (2.36)	435.5	0.53	0.09
MBEA contour (/30)	25.07 (2.59)	25.10 (3.20)	459.0	0.77	0.04
MBEA interval (/30)	24.77 (2.79)	24.74 (3.13)	475.5	0.95	0.01
MBEA pitch composite (/90)	75.87 (6.65)	76.23 (7.34)	459.0	0.77	0.04
Digit span	7.16 (1.61)	7.13 (1.09)	467.0	0.85	0.03
Autistic traits (AQ)	34.81 (8.24)	16.77 (7.86)	898.0	< 0.01	0.87

*Note*: Maximum possible scores are indicated with a slash (e.g., /60 for RSPM and /30 for MBEA subtests). Both raw and standard scores are provided for RSPM and ROWPVT‐4. All other scores are reported as raw values.

Abbreviations: MBEA = Montreal Battery of Evaluation of Amusia; ROWPVT‐4 = Receptive One‐Word Picture Vocabulary Test, Fourth Edition; RSPM = Raven's Standard Progressive Matrices.

The study received ethical approval from the University Research Ethics Committee, and all participants provided written informed consent. Participants were financially compensated and reimbursed for travel expenses. Students from the human participant pool received course credit for their involvement.

### 2.2 Stimuli and Apparatus

The stimuli and methods were adapted from Patel et al.'s (1998) design, utilizing de Leeuw et al.'s (2019) streamlined approach to reduce the total number of stimuli to 172 (100 sentences and 72 musical excerpts) while preserving critical contrasts. The P600 component was evaluated by comparing ERP amplitudes between grammatically incorrect and correct sentences in the language condition, as well as between in‐key and out‐of‐key musical excerpts in the music condition.

Language stimuli consisted of sentences lasting 3–4 s, spoken at a rate of approximately six syllables per second. To elicit the P600 effect, each sentence contained a target noun phrase (in bold font in examples below) that was either syntactically congruent (grammatical) or incongruent (ungrammatical) with the preceding sentence context, as illustrated in examples (a) and (b).

To prevent reliance solely on local context (underlined in examples), two types of fillers were included, one for each condition. Fillers for the grammatical condition were grammatically correct but conceptually unacceptable, presenting cases where the target noun phrase following “had” was not always acceptable. Fillers for the ungrammatical condition were grammatically correct, including instances where verb and “the” combinations were not always acceptable.

- Grammatical: One of the directors had filmed **a beautiful shot of the sunset**.
- Ungrammatical: One of the artists ignored the filmed **a beautiful shot of the sunset**.
- Filler for (a): One of the photographers had convinced **a beautiful shot of the sunset**.
- Filler for (b): One of the artists ignored the essay on **a beautiful shot of the sunset**.

**FIGURE 1:**
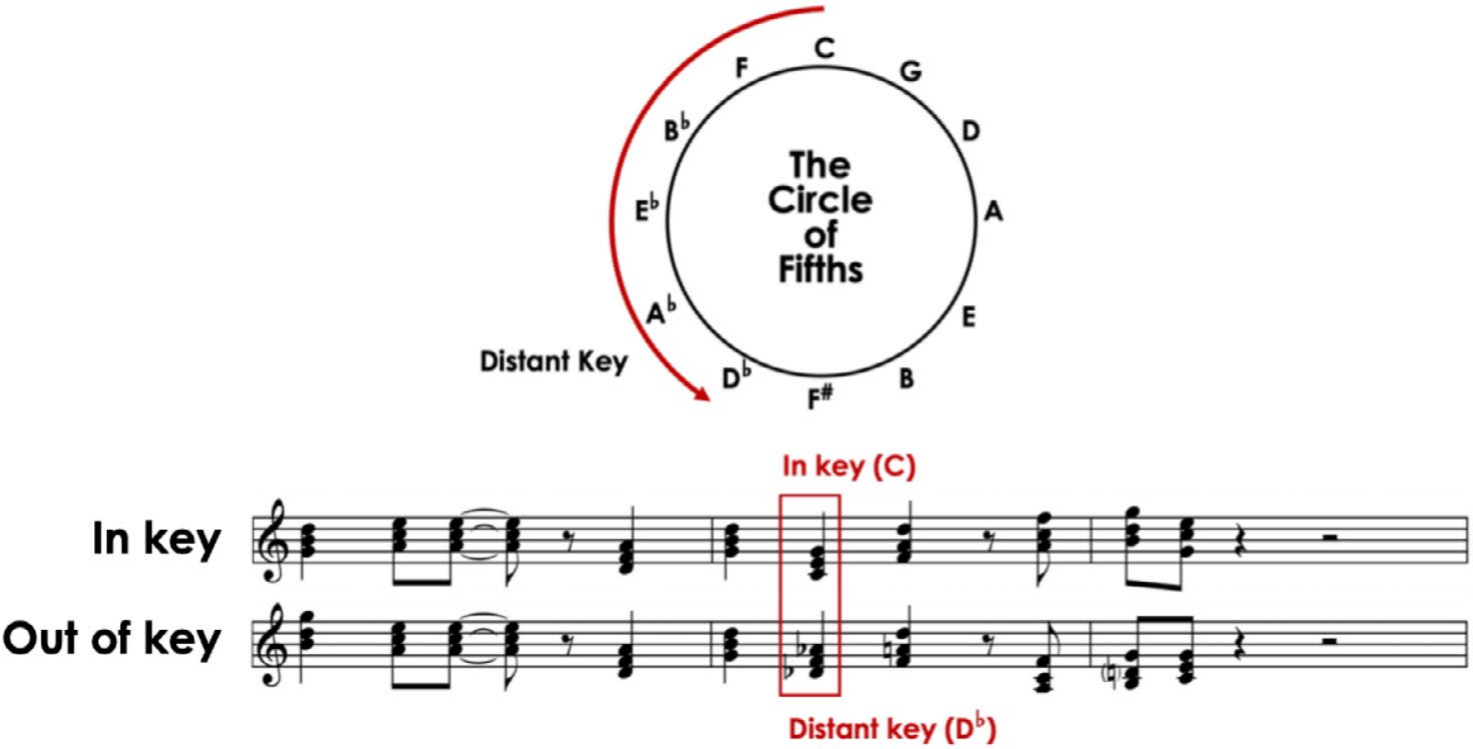
Example of musical phrase pairs adapted from Patel et al. (1998). Both sequences begin with chords in key (C major), establishing harmonic expectancy. In the out‐of‐key (ungrammatical) condition, a syntactic violation occurs at the marked section where the progression shifts unexpectedly to a distant key (D^♭^ major), disrupting harmonic expectancy. The circle of fifths illustrates the tonal distance between C major and D^♭^ major.

Musical stimuli consisted of short sequences of 7–12 chords, each lasting approximately 6 s, with chords occurring at a rate of about 1.8 per second. The acceptability of music was manipulated using the circle of fifths, a fundamental concept in Western tonal music theory that describes relationships between keys. Chords from more distant keys on the circle sound inharmonious when combined (Bharucha and Stoeckig 1986). In both conditions, chord sequences initially followed harmonic expectations, establishing a coherent musical structure before the appearance of the target chord. In the in‐key condition (acceptable), all chords remained within the same key, preserving harmonic stability. In contrast, in the out‐of‐key condition (unacceptable), the target chord was replaced with a distant chord (see Figure 1 for an example), creating a sound inharmonious to the preceding melody.

Each pair of in‐key and out‐of‐key chords were matched for length, rhythm, and chord count, ensuring consistency in the acoustic context. This design emphasizes structural harmonic processing, requiring the integration of incoming chords within an established musical framework. All stimuli were normalized to 74 dB for consistent volume levels.

### 2.3 Procedure

Participants sat in a sound‐proof booth in front of a monitor and keyboard. Stimuli were presented via E‐Prime 3.0 (Psychology Software Tools, 2016), with audio delivered through Etymotic ER‐1 insert earphones using an RME Fireface UC sound card. The task involved judging the acceptability of sentences and musical sequences. For language, acceptability was determined by whether sentences followed grammatical and semantic rules. For music, participants judged whether sequences sounded natural or unexpected based on their implicit musical intuition. Participants completed practice trials (six language and six music items) before the main experiment to ensure task understanding, with instructions repeated if needed. No feedback was provided during practice or the experiment. This approach ensures that participants rely on their natural intuitions rather than external corrections. This is particularly important for the music condition, where participants without formal training might lack explicit rule‐based knowledge. Providing feedback could bias their judgments or introduce learning effects.

Each trial began with an audio file and a fixation cross, followed by a 1450 ms blank screen. Participants then judged acceptability by pressing “C” or “M” (key assignments counterbalanced). The experiment had five blocks: three language blocks (33, 33, and 34 trials) and two music blocks (36 trials each), alternating between language and music. Blocks included randomized acceptable and unacceptable trials, with self‐paced breaks to reduce fatigue.

### 2.4 EEG Recording and Pre‐Processing

EEG data were collected using a Biosemi Active Two system equipped with 64Ag/AgCl electrodes on an elastic cap and six external electrodes (left and right mastoids, two vertical, and two horizontal electrooculography electrodes). EEG signals were recorded at a sampling rate of 2048 Hz without referencing, with electrode impedances maintained below 25 kΩ. Time‐aligned triggers marking the onset of the target tone or word were captured.

EEG pre‐processing was performed in MATLAB R2018b using EEGLAB (Delorme and Makeig 2004). Data were re‐referenced to the mastoids, filtered (0.1–30 Hz), downsampled to 500 Hz, and segmented into epochs from 100 ms pre‐ to 1000 ms post‐target onset with baseline correction. Bad channels were interpolated, and independent component analysis (Infomax algorithm) was used to remove ocular and muscle artifacts. Across participants, an average of 0.98 channels were interpolated, with a maximum of four channels per participant. Interpolation was applied in 33 out of 62 participants. Trials exceeding ±150 μV were discarded, with an average of 2.85 trials per participant (SD = 3.30).

### 2.5 Data Analysis

Cluster‐based permutation tests (Maris and Oostenveld 2007) were conducted using Fieldtrip (Oostenveld et al. 2011) to identify significant P600 effects across groups and conditions. Paired *t*‐tests compared grammatical vs. ungrammatical conditions at each electrode and time point (500–1000 ms). *T* values exceeding *p* < 0.05 were clustered by neighboring time points and electrodes, with significance determined via Monte Carlo simulations (1000 permutations). Clusters were significant if their statistics fell within the top or bottom 2.5th percentile (two‐tailed *p* = 0.05).

Since the cluster‐based permutation test only allows comparison between two conditions at a time, linear mixed effects (LME) analyses were conducted to examine interactions. Statistical analyses were conducted in R v4.4.1 (R Core Team 2024) using the lme4 package (Bates et al. 2015). LME models analyzed grand‐averaged P600 amplitudes, while generalized linear mixed effects (GLME) models assessed binary accuracy data. Fixed effects included Group (Autistic vs. Non‐autistic), Condition (Language vs. Music), and their interaction. Random effects comprised by‐subject intercepts and slopes for Condition, with an additional by‐item intercept for behavioral data; by‐item slopes were excluded due to convergence issues. Fixed effect significance was assessed using likelihood ratio tests, with Bonferroni corrections for multiple comparisons.

## 3 Results

### 3.1 Behavioral Results

**FIGURE 2:**
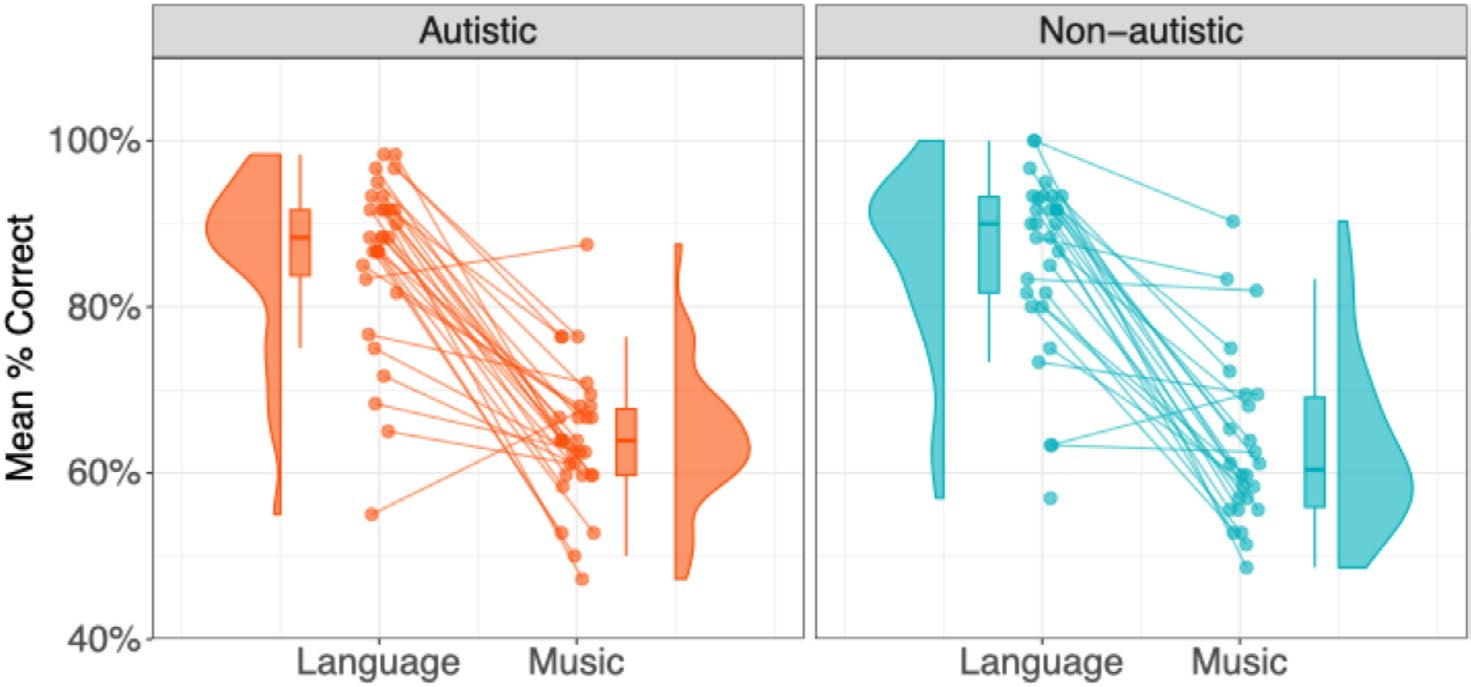
Behavioral accuracy across groups and conditions. Each panel shows a violin plot with a box plot, displaying the distribution of mean percentage correct responses. Lines connect individual data points to highlight within‐subject differences.

Figure 2 illustrates the behavioral accuracy results across conditions and groups, including individual data distributions. In the language condition, the non‐autistic group exhibited a narrower data spread, indicating more consistent performance, while the autistic group displayed a broader spread. No such distribution difference was observed in the music condition. GLME models (see Table 2) revealed a significant main effect of condition, indicating higher accuracy in the language condition (Non‐autistic: Mean = 86.7%, SD = 34.0%; Autistic: Mean = 84.1%, SD = 36.6%) compared to the music condition (Non‐autistic: Mean = 63.8%, SD = 48.1%; Autistic: Mean = 64.4%, SD = 47.7%). However, no significant group effect or interaction was observed, suggesting no overall performance difference between the autistic and non‐autistic groups.

**TABLE 2:** Results of the GLME models for behavioral accuracy data.

Fixed effects	Est/beta	SE	*z*	*χ*2	*p*
(Intercept)	1.50	0.07	17.88	—	—
Group	−0.07	0.14	−0.46	0.21	0.644
Condition	1.38	0.11	12.73	83.18	< 0.001
Group × condition interaction	−0.18	0.21	−0.85	0.72	0.398

Table 3 presents the accuracy across conditions in our study, alongside the results from Patel et al. (1998) and de Leeuw et al. (2019) for comparison. Overall, our results replicated previous findings, showing higher accuracy in the language condition compared to the music condition. However, accuracy in our study was lower across both groups, with greater variability, particularly in the music condition. This may be due to differences in participants' musical backgrounds; our sample included individuals with varying levels of musical experience, whereas previous studies focused on participants with formal musical training.

**TABLE 3:** Comparison of behavioral accuracy across language and music conditions in the current study and previous studies.

Condition	Patel et al. (1998)	de Leeuw et al. (2019)	Current study	
			NAS	AS
Language, grammatical	*M* = 95%	*M* = 93.3% SD = 5.3%	*M* = 89.6% SD = 30.4%	*M* = 88.6% SD = 31.8%
Language, ungrammatical	*M* = 96%	*M* = 88.2% SD = 18.0%	*M* = 80.1% SD = 39.9%	*M* = 83.7% SD = 37.0%
Music, in‐key	*M* = 80%	*M* = 84.5% SD = 14.5%	*M* = 70.1% SD = 45.9%	*M* = 70.9% SD = 45.5%
Music, out‐of‐key	*M* = 72%	*M* = 69.1% SD = 15.5%	*M* = 57.4% SD = 49.5%	*M* = 58.0% SD = 49.4%

A secondary analysis was conducted excluding four participants (three autistic and one non‐autistic), whose MBEA pitch composite scores were below the threshold of 65, indicative of potentially having amusia (Liu et al. 2010). The results were consistent with the initial analysis showing higher accuracy in the language condition compared to the music condition in both groups (see details in Supporting Information S1).

Additionally, to account for participants' response bias to grammatical and ungrammatical trials, we calculated the hit rate and false alarm rate for each group and condition. No significant group differences were found, indicating no response bias between groups (see details in Supporting Information S1).

### 3.2 ERP Results

**FIGURE 3:**
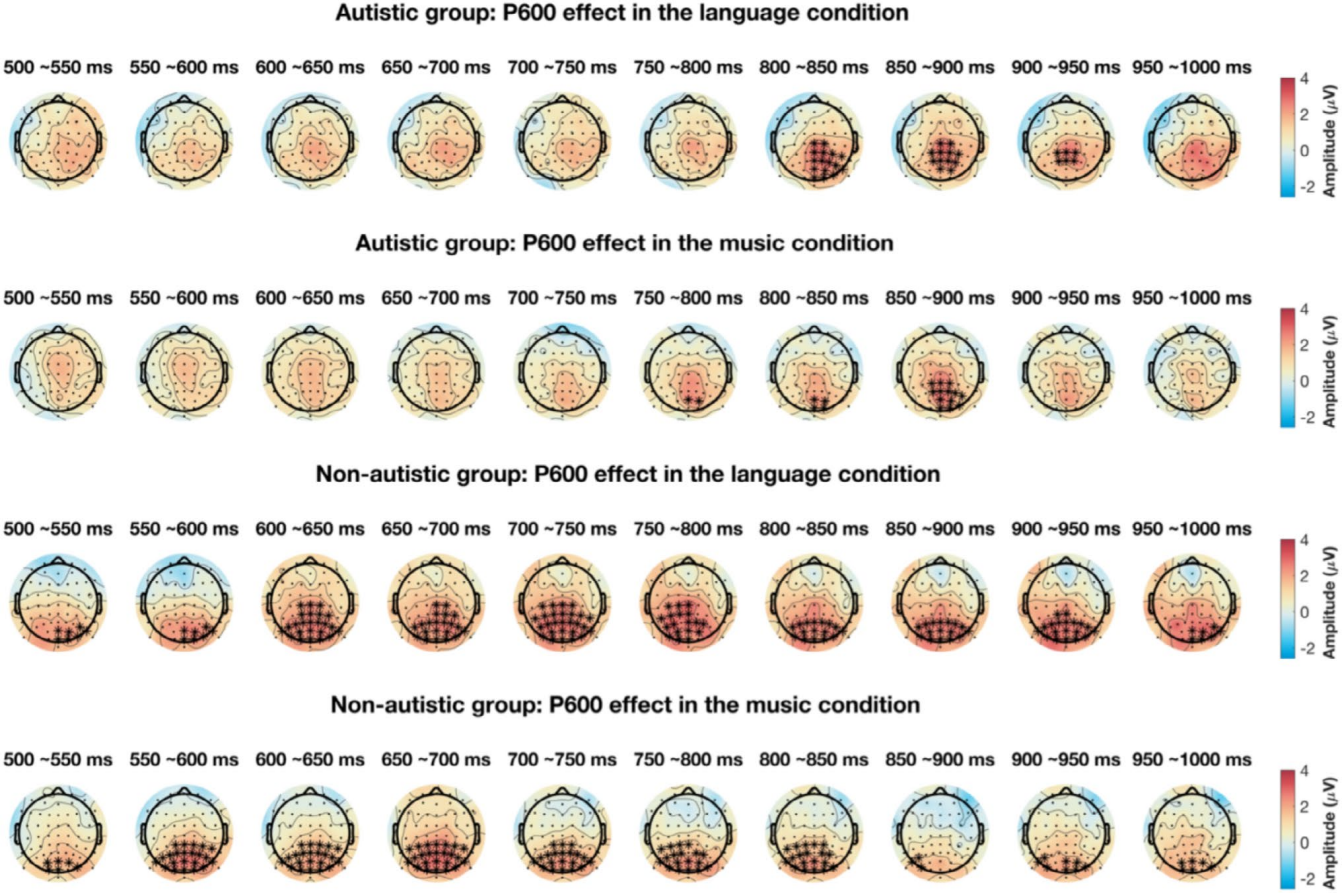
Topographic maps showing *t*‐values of each sample pair (time‐electrode) in the permutation analyses, comparing grammatical vs. ungrammatical trials (i.e., P600 effect) across conditions and groups. Significant clusters are marked with “*.”

Cluster‐based permutation tests revealed amplitude differences between grammatical and ungrammatical conditions (i.e., the P600 effect) within the 500–1000 ms window for both groups, as shown in Figure 3. Non‐autistic participants exhibited pronounced effects in the central and posterior regions starting at 500 ms for both language (*p* = 0.002) and music conditions (*p* = 0.002). In contrast, autistic participants showed delayed effects, emerging between 800 and 950 ms for language (*p* = 0.006) and between 750 and 900 ms for music (*p* = 0.02), with distributions more localized to midline regions, indicating longer latency and atypical topography compared to the non‐autistic group.

**FIGURE 4:**
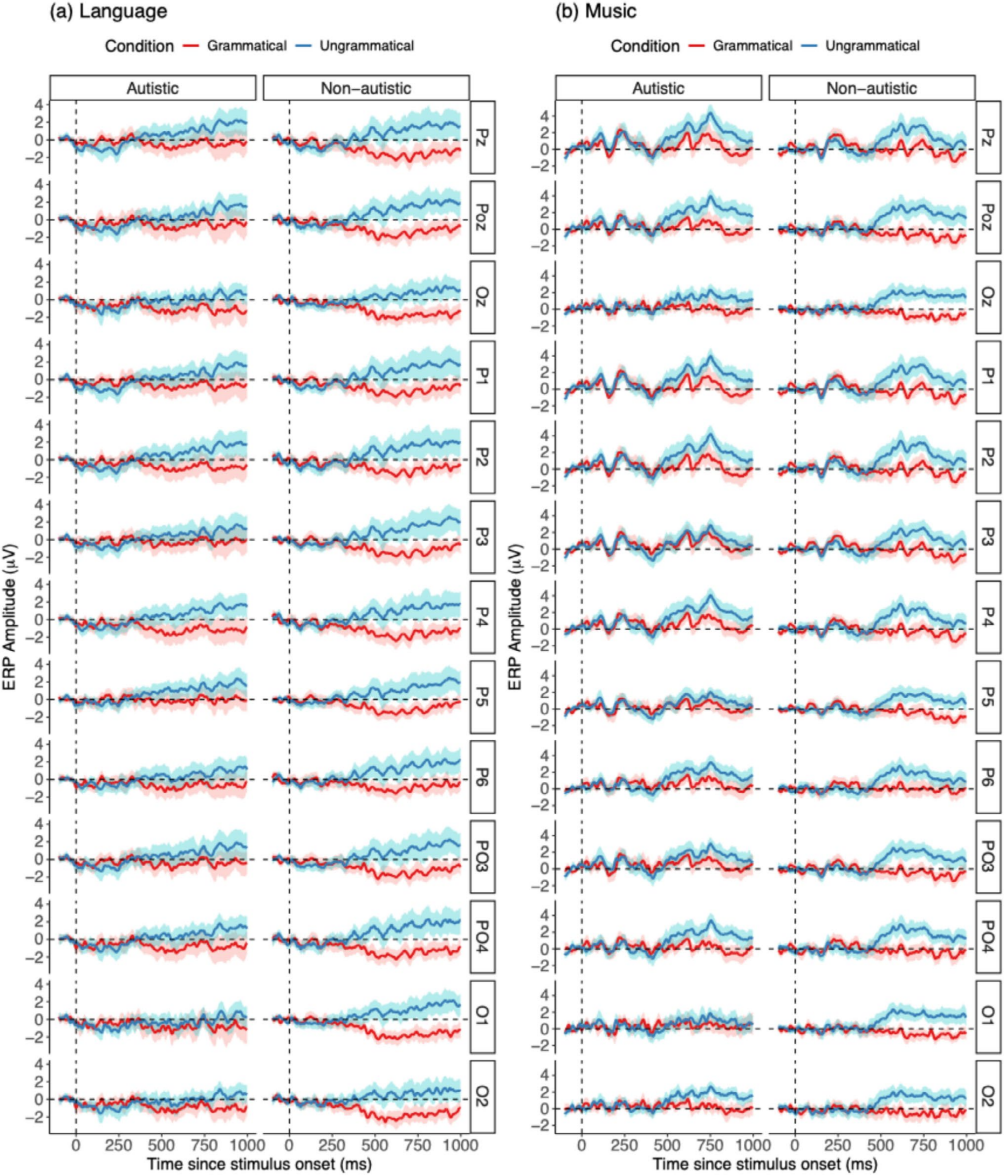
ERP waveforms for P600 at posterior electrode sites for (a) language and (b) music stimuli in both groups. The red lines depict responses to grammatical stimuli, while the blue lines represent responses to ungrammatical stimuli. Shaded areas represent 95% confidence intervals. Electrode labels (e.g., Pz and POz) are in the right‐hand boxes. Time is on the *x*‐axis, and ERP amplitude is on the *y*‐axis.

To compare language and music conditions within each group, additional tests were performed, revealing no notable differences between grammatical and ungrammatical conditions, suggesting similar P600 effects across both domains. We constructed an LME model to examine P600 amplitude differences across conditions and groups within the 500–800 ms time window, as identified by de Leeuw et al. (2019) for P600 analysis. Amplitudes from posterior electrodes where P600 activity typically occurs (Wang, Ong, et al. 2023; Wang, Schoot, et al. 2023) were selected as the outcome measure. The model revealed a group effect, with non‐autistic participants displaying larger P600 amplitudes than autistic participants (see Table 4 for results). No condition effects or interactions were found. ERP waveforms across conditions are shown in Figure 4.

**TABLE 4:** Results of the LME models for ERP data.

Fixed effects	Est/beta	SE	*t*	*χ*2	*p*
(Intercept)	1.86	0.30	6.28	—	—
Group	−1.23	0.59	−2.08	4.18	0.041
Condition	0.23	0.54	0.52	0.19	0.665
Group × condition interaction	−0.50	1.08	−0.46	0.21	0.643

Overall, our results indicated a delayed and reduced P600 in autistic participants. To investigate whether this reduction was associated with lower‐level acoustic processing, we examined the N1‐P2 components for target chords in the music condition. The rationale for restricting this analysis to music stems from the distinct temporal properties of linguistic and musical stimuli. In music, each chord was separated by approximately 20 ms of silence, allowing the N1‐P2 complex to emerge as a distinct auditory response around 100 ms after target onset. In contrast, speech stimuli lacked comparable silent intervals, preventing the isolation of a clear N1‐P2 response. As a result, N1‐P2 analysis was only feasible for the music condition. ERP waveforms for the earlier time window in both conditions are shown in Figure S2, clearly illustrating the absence of an N1‐P2 response in the language condition, consistent with Patel et al. (1998). Both permutation tests and ANOVAs revealed no significant group differences, suggesting similar acoustic processing in the music condition across groups (see results in Supporting Information S1).

Additionally, we examined early ERP components typically associated with syntactic violations: the Early Right Anterior Negativity (ERAN) for musical syntax and the Left Anterior Negativity (LAN) for linguistic syntax. However, no significant ERAN or LAN responses to the syntactic violations were observed in either group (see Supporting Information S1 for details).

### 3.3 Correlations

We examined whether demographic and cognitive factors influenced performance using Pearson correlations between behavioral accuracy, P600 amplitude, and various demographic and cognitive variables. To control for multiple comparisons, a Bonferroni correction was applied. After correction, none of the correlations remained statistically significant, indicating no significant correlations between participants' behavioral and neural performance and their demographic and cognitive abilities (see correlation matrix in Figure S5).

## 4 Discussion

This study investigated behavioral and neurophysiological responses to syntactic processing in language and music among autistic and non‐autistic adults matched for demographic and cognitive characteristics. Behaviorally, both groups demonstrated comparable accuracy when identifying syntactic violations, with higher accuracy in the language condition than in the music condition, which replicated previous research (see Table 3). Neurophysiologically, both groups exhibited similar P600 responses to linguistic and musical syntax, demonstrating “statistically indistinguishable” syntactic integration across domains. However, group differences emerged in P600 responses. Non‐autistic participants exhibited a P600 starting at 500 ms across posterior regions, consistent with the patterns observed in Patel et al. (1998) and de Leeuw et al. (2019). In contrast, the P600 effect in the autistic group showed longer latency, reduced amplitudes, and a more restricted distribution. The absence of a group × condition interaction suggests that autistic participants displayed similarly atypical neural processing of syntax for both language and music. This provides the first evidence of parallel syntactic processing difficulties across both domains in autism, supporting the SSIRH.

The discrepancy between behavioral and ERP results in autistic individuals highlights how different methodological approaches capture distinct aspects of syntactic processing and underscores the value of electrophysiological methods in uncovering real‐time neural mechanisms. In the acceptability judgment task, the autistic participants were as accurate as non‐autistic participants, which demonstrated their intact sensitivity to offline syntactic information (Heaton et al. 2007; Janke and Perovic 2015). However, they exhibited less efficient syntactic processing and a more restricted neural activation pattern, as indicated by the delayed and attenuated P600 responses during rapid, online integration (Hagoort et al. 1993). In our task, syntactically unexpected target words or musical chords appeared later in the sequences, requiring participants to generate predictions based on syntactic rules while processing earlier elements. The increased P600 response to these incongruent elements indicated an escalating cognitive demand for structural resolution (Friederici 2002; Hagoort 2003). For autistic participants, these demands may have exceeded available cognitive resources, leading to reduced and delayed P600 effects. Therefore, this pattern likely reflects inefficient resource allocation for syntactic integration and/or restricted processing capacities (Kaan et al. 2000; Regel et al. 2014).

It should be noted that the ERP waveforms for linguistic and musical stimuli differed in shape. In the music condition, a downward deflection was observed after 500 ms, whereas no comparable deflection appeared in the language condition. This pattern is in line with findings from Patel et al. (1998), which attributed it to the N1‐P2 complex elicited by the onset of the chord following the target chord. According to Patel et al. (1998), given that chords were presented at a rate of ~1.8 per second (i.e., a new chord every ~500 ms), the onset of each subsequent chord fell within the ERP time window of the preceding chord. As a result, the N1‐P2 response to the next chord would emerge after 500 ms, aligning with the observed deflection. Supporting this interpretation, the deflection was visible in posterior regions (Figure 4) but was more pronounced and followed a clearer pattern in anterior regions, where the N1‐P2 complex is typically strongest (Figure S2).

This study extends the application of the SSIRH to autism, building on prior ERP research that has examined syntactic integration in other atypical populations, including aphasia, amusia, and DLD. However, findings across these populations vary. In aphasia, Chiappetta et al. (2022) reported an impaired P600 in the language condition but not in the music condition, suggesting a dissociation rather than a shared syntactic processing deficit. This aligns with Slevc et al. (2016), who found that language impairments do not always extend to music. In contrast, congenital amusia, a neurodevelopmental disorder affecting pitch perception, has been linked to P600 impairments in both music and language (e.g., Sun et al. 2018), which supports the SSIRH. Similarly, studies in children with DLD (Jentschke et al. 2008) found parallel syntactic difficulties in both domains, reinforcing the idea that music and language share common syntactic integration mechanisms.

One explanation for these differences may relate to distinct neural mechanisms underlying syntactic processing across these populations. Aphasia and acquired amusia are typically caused by focal brain lesions, leading to selective impairments in syntactic integration for language/music, while DLD, congenital amusia, and autism are neurodevelopmental conditions characterized by altered structural and functional connectivity. From this perspective, DLD may be more comparable to autism, as both conditions exhibit atypical patterns of brain connectivity, particularly in regions associated with language processing (Krishnan et al. 2016). Future research should explore how differences in functional connectivity in autism influence syntactic processing across language and music.

The cross‐domain syntactic integration difficulties observed in our study contribute to the ongoing debate on whether autistic individuals show a dissociation between language and music processing. Some research suggests intact musical abilities despite linguistic challenges at the acoustic level (DePriest et al. 2017; Jiang et al. 2015; Lai et al. 2012; Sharda et al. 2015), while other studies report parallel performance in higher‐level cognitive processing across both domains (Wang, Ong, et al. 2023; Wang, Schoot, et al. 2023; Zhao et al. 2024). By specifically examining syntactic processing, our findings reveal that autistic individuals struggle with rapid syntactic integration in both language and music, providing evidence for a shared underlying mechanism.

A local processing bias, as described in the weak central coherence (WCC) theory, may underlie cross‐domain syntactic integration difficulties in autism. According to this theory, autistic individuals tend to prioritize local detail processing at the expense of global integration (Frith 1989; Frith and Happé 1994). This bias can affect both linguistic and musical syntax, as both require the building of a hierarchical structure beyond immediate local dependencies. Syntactic integration involves more than recognizing individual words or chords; it requires establishing how those elements fit into the overall grammatical structure (Deutsch 1999; Lerdahl 2005).

In the present study, linguistic violations involved phrase structure errors, where a word's syntactic role could not be correctly assigned within the expected sentence structure. Similarly, musical violations involved harmonic progressions that, while locally acceptable at the chord level, disrupted the overall coherence of the music sequence. In both cases, efficient detection of syntactic violations, as reflected in the P600 effect, required participants to process structural relationships beyond adjacent elements, integrating local components into a global syntactic framework.

Due to their local processing tendency, autistic individuals may focus more on local lexical features in language and isolated tones in music rather than integrating these elements into broader structures. This difference in processing hierarchical relationships may make it more challenging to detect phrase structure violations in language and harmonic irregularities in music, as reflected by reduced P600 responses across domains. Our results suggest that these differences in syntactic processing are not confined to language but extend across domains. This aligns with the predictions of the SSIRH, which proposes that syntactic processing in language and music relies on overlapping neural resources.

Our findings also contribute to a longstanding debate within the WCC framework: whether integration difficulties result from poor structural knowledge (e.g., language impairments) or reflect a distinct processing style (Brock et al. 2008; Snowling and Frith 1986). Although the two groups in our study were matched on language and musical abilities, and performed similarly in detecting syntactic violations, autistic participants showed inefficient neural responses during syntactic integration. This implies that WCC reflects a cross‐domain cognitive style in autism rather than reduced structural knowledge. Another debated point in WCC is whether it indicates genuine global integration difficulties or an enhanced ability to focus on local details (Bojda et al. 2021). Our analysis of early ERP components (N1‐P2) in response to acoustic processing found no significant group differences in the music condition, indicating no heightened local acoustic processing in autism.

This study has a few limitations that should be considered. First, the ERP analysis relied on event‐related averaging, isolating neural responses to specific events like word or chord onsets. This method requires short intervals between stimuli to ensure a clear baseline and well‐defined responses. However, acoustic differences between language and music stimuli posed challenges for comparable analyses across domains. For example, the brief intervals in the music condition allowed the N1‐P2 complex to reflect local acoustic features, while the continuous nature of language stimuli precluded similar analysis. Consequently, direct comparisons of auditory processing across domains were not possible. Future studies should employ advanced neural tracking techniques to model brain responses to continuous auditory input, offering insights into multi‐level processing. Additionally, as Featherstone et al. (2013) noted, musical violations can vary in probability and adherence to Western harmonic rules, potentially introducing confounds. While this study replicated Patel et al.'s (1998) tasks and supported their findings, future research should expand the framework with diverse stimulus types and designs to enhance robustness.

Another limitation concerns participants' musical backgrounds, which likely influenced performance in the music condition. Unlike earlier studies with participants averaging 11 years of musical training (Patel et al. 1998) or a minimum of 4 years (de Leeuw et al. 2019), our participants had varied musical experiences (see Table 1). This variability may explain the lower accuracy observed in the music condition. Future studies should explore how formal musical training influences syntactic processing in autism.

Additionally, focusing on a specific subgroup of autistic individuals matched with non‐autistic peers limits the generalizability of our findings. Future research should explore syntactic processing across a broader range of autistic individuals with diverse cognitive and language abilities.

Finally, syntactic integration involves interactions between frontal and posterior brain regions (Patel 2003, 2010). According to the SSIRH, shared neural resources facilitating syntactic integration are located in frontal areas, while domain‐specific syntactic representations reside in posterior regions (Haarmann and Kolk 1991; Kaan and Swaab 2002). Although our findings associate deficient syntactic processing in autism with atypical posterior P600 activity, it remains unclear whether compensatory mechanisms or alternative neural pathways also play a role in their atypical processing. Future neuroimaging studies are needed to provide a more comprehensive understanding of the neural basis of syntactic integration in autism.

## 5 Conclusion

This study investigated syntactic processing in both language and music among autistic and non‐autistic adults using behavioral and neural measures. Despite intact behavioral performance, autistic individuals exhibited atypical P600 responses across both domains, suggesting domain‐general difficulties in syntactic integration, potentially linked to WCC in autism. Our findings support the SSIRH in autistic individuals with intact cognitive abilities, marking the first test of this hypothesis in autism. This study offers insights into the neural mechanisms underlying syntactic processing in autism.

## Conflicts of Interest

The authors declare no conflicts of interest.

## Supporting information


**Data S1.** Supporting Information.

## Data Availability

The data that support the findings of this study are available on request from the corresponding author. The data are not publicly available due to privacy or ethical restrictions.
