# Supplementary material for: Linguistic and Musical Syntax Processing in Autistic and Non‐Autistic Individuals: An Event‐Related Potential (ERP) Study
Source: Autism Res. 2025 Apr 5;18(6):1245–56. doi: 10.1002/aur.70038 (PMC12166516; doi:10.1002/aur.70038)
Supplement: Supplementary file 1 — Data S1. Supporting Information. [file AUR-18-1245-s001.docx]

**Linguistic and musical syntax processing in autistic and non-autistic individuals: An event-related potential (ERP) study**

**Supplementary Material**

**1. Behavioural analysis**

**1.1 Accuracy analysis excluding outliers**

A secondary analysis was conducted excluding four participants (three autistic and one non-autistic), whose Montreal Battery of Evaluation of Amusia (MBEA) pitch composite scores were below the threshold of 65. Supplementary **Figure 1** illustrates the behavioural accuracy results across conditions and groups, including individual data distributions. LME models (see Supplementary Table 1) revealed a significant main effect of condition, indicating higher accuracy in the language condition (Non-autistic: Mean = 86.7%, SD = 34.0%; Autistic: Mean = 86.0%, SD = 34.8%) compared to the music condition (Non-autistic: Mean = 63.8%, SD = 48.1%; Autistic: Mean = 64.5%, SD = 47.9%). However, no significant group effect or interaction was observed, suggesting no overall performance difference between the autistic and non-autistic groups.

**Supplementary Figure 1**. Performance accuracy excluding participants with MBEA scores below 65 in each group and condition. Each panel displays a violin plot with an embedded box plot, indicating the distribution of mean percentage correct responses. Individual participant data points are connected by lines to illustrate within-subject differences.


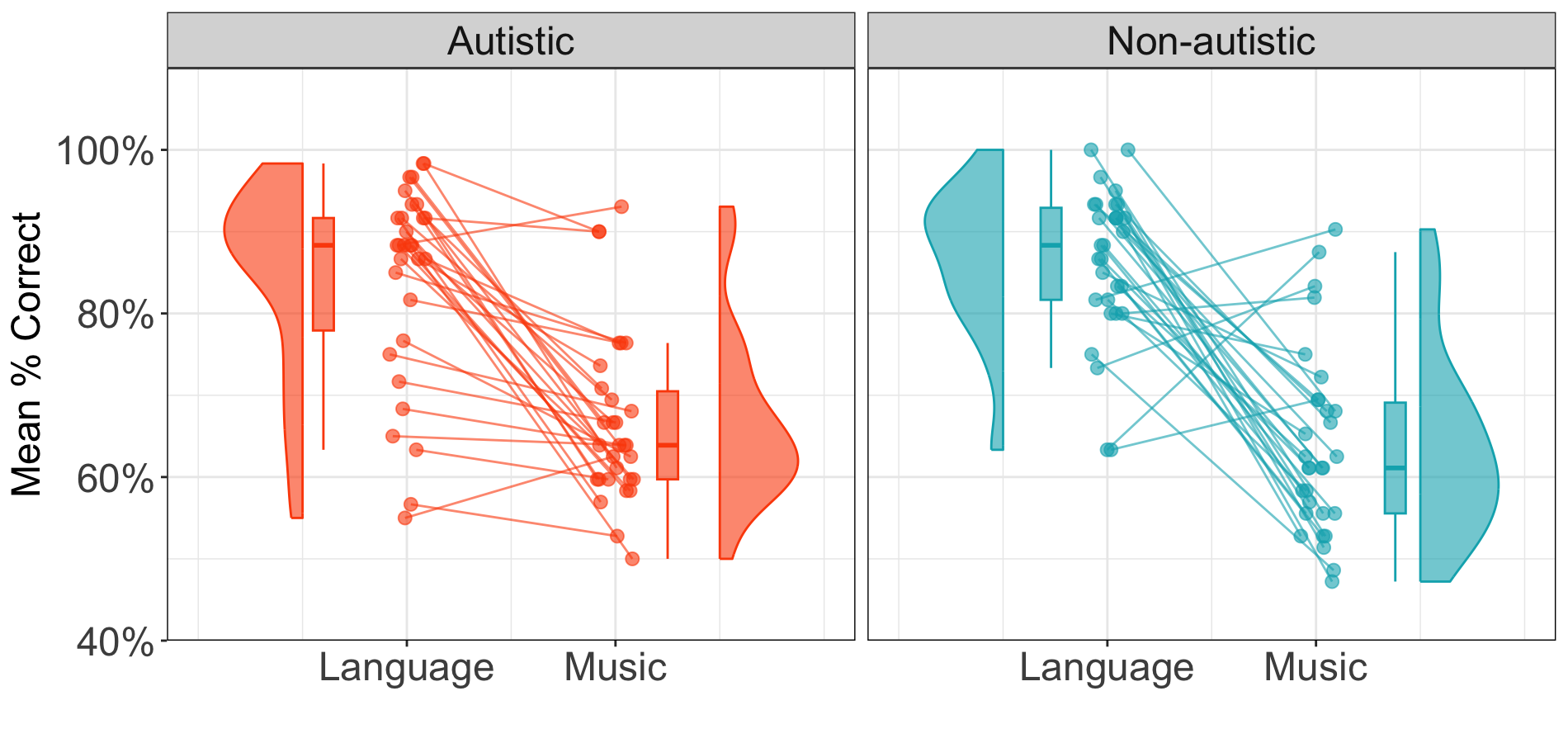


**Supplementary Table 1.** Results of the GLME models for behavioural accuracy data.

| **Fixed effects** | **Est/Beta** | **SE** | **z** | ***χ2*** | ***p*** |
| --- | --- | --- | --- | --- | --- |
| (Intercept) | 1.53 | 0.12 | 13.25 | — | — |
| Group (AS vs. NAS) | -0.02 | 0.16 | -0.14 | 0.02 | 0.887 |
| Condition (Language vs. Music) | 1.65 | 0.20 | 8.09 | 57.66 | **<0.001** |
| Group × Condition Interaction | -0.09 | 0.24 | -0.41 | 0.162 | 0.688 |

**1.2 Response bias**

To examine participants’ sensitivity in judging the acceptability of the sentences while accounting for response bias, we computed d-prime (d') using the psycho package in Rstudio (Makowski, 2018). Hit rates were defined as correctly identifying ungrammatical sentences, while false alarms referred to incorrectly classifying grammatical sentences as ungrammatical. A higher d' value indicates greater sensitivity in judging sentence acceptability, reflecting the ability to correctly identify both grammatical and ungrammatical sentences while accounting for response bias. To avoid extreme values (e.g., hits = 100% or 0%) we applied the adjusted SDT correction when computing d' (Hautus, 1995).

A 2 × 2 mixed-design ANOVA was conducted for d', with group (autistic, non-autistic) as a between-subjects factor and condition (language, music) as a within-subjects factor. The analysis revealed a significant main effect of condition, *F*(1, 60) = 181.14, *p* < .001, *η²p* = 0.51, indicating that d' value was significant higher in the language (*M* = 2.37, *SD* = 0.89) condition, compared to the music (*M* = 0.81, *SD* = 0.63) condition. However, there was no significant main effect of group, *F*(1, 60) = 0.87, *p* = .356, suggesting that autistic and non-autistic participants had comparable sensitivity to grammatical violations across conditions. The group × condition interaction was also not significant, *F*(1, 60) = 1.32, *p* = .255, indicating that the effect of condition did not differ between groups.

**2. N1-P2 Analyses**

Cluster-based permutation analyses were performed, following a procedure similar to the P600 effect analysis described earlier. ERP amplitudes from frontal-central electrodes (AFz, C3, C4, Cz, F1, F2, F3, F4, FC1, FC2, FC3, FC4, FCz, FPz, Fz) were analysed within the 0-300ms time window post-target onset, focusing on the music condition. Two between-group contrasts were examined: one comparing autistic and non-autistic participants in the grammatical condition and the other in the ungrammatical condition. These analyses aimed to identify significant clusters of time points and electrode sites that might reveal group differences in early acoustic processing across syntactic conditions. No significant clusters were found in either contrast, indicating no significant group differences in ERP amplitude within the 0-300ms window for both grammatical and ungrammatical conditions.

Additionally, we conducted two-way ANOVAs to analyse the peak amplitudes of the N1 and P2 components in the frontal-central regions. ERP waveforms within the 0-300ms window were grand-averaged across groups and conditions to establish the latency of the grand mean N1 and P2 components. These grand mean latency values were then used to select the N1 and P2 peak amplitudes for each individual participant, using a window of ±20ms around the grand mean latency (see McClannahan et al., 2019 for a similar method). The peak amplitudes were subsequently used in the ANOVA analyses. Supplementary Table 2 presents the results of these analyses, which reveal no significant main effects or interactions for either N1 or P2 amplitudes. This suggests that comparable N1-P2 responses were elicited by the acoustic properties of the music stimuli in both autistic and non-autistic groups (see also Supplementary Figure 2 & 3 for ERP wave forms and grand average amplitude).

**Supplementary Table 2.** The results of ANOVAs examining differences in N1-P2 amplitudes between groups (autistic vs. non-autistic) and across conditions (grammatical vs. ungrammatical).

| ERP Components | **Factor** | | |
| --- | --- | --- | --- |
|  | **Group** | **Condition** | **Group x Condition** |
| N1 | F(1, 60) = 0.02  *p* = 0.892 | F(1, 60) = 0.35  *p* = 0.557 | F(1, 60) = 1.07  *p* = 0.304 |
| P2 | F(1, 60) = 0.01  *p* = 0.905 | F(1, 60) = 1.19  *p* = 0.280 | F(1, 60) = 0.08  *p* = 0.782 |

**Supplementary Figure 2.** ERP waveforms for the N1-P2 responses at frontal-central electrode sites for (a) language and (b) music stimuli in both groups. The green lines depict responses to grammatical stimuli, while the orange lines represent responses to ungrammatical stimuli. Shaded areas indicate 95% confidence intervals. Electrode labels (e.g., AFz) are shown in the right-hand boxes. Time is on the x-axis, and ERP amplitude is on the y-axis. A clear N1-P2 complex is present in the music condition for both groups, whereas no observable N1-P2 response is evident in the language condition.


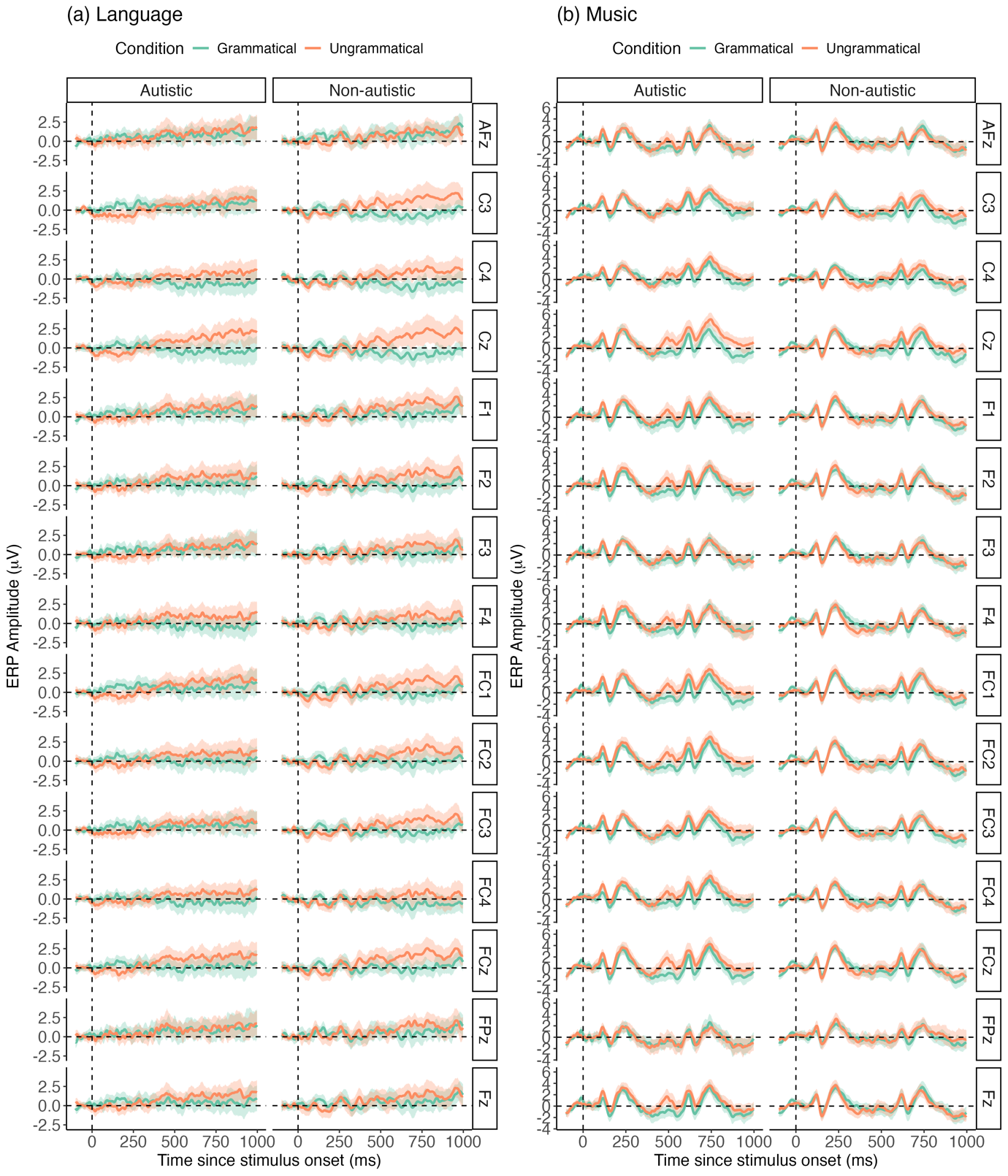


**Supplementary Figure 3.** ERP waveforms for the N1-P2 responses in the 0-300ms time window across frontal-central electrode sites elicited by the music condition in both groups. The green lines depict responses in the autistic (AS) group, while the orange lines represent responses in the non-autistic (NAS) group. Shaded indicate the 95% confidence intervals.


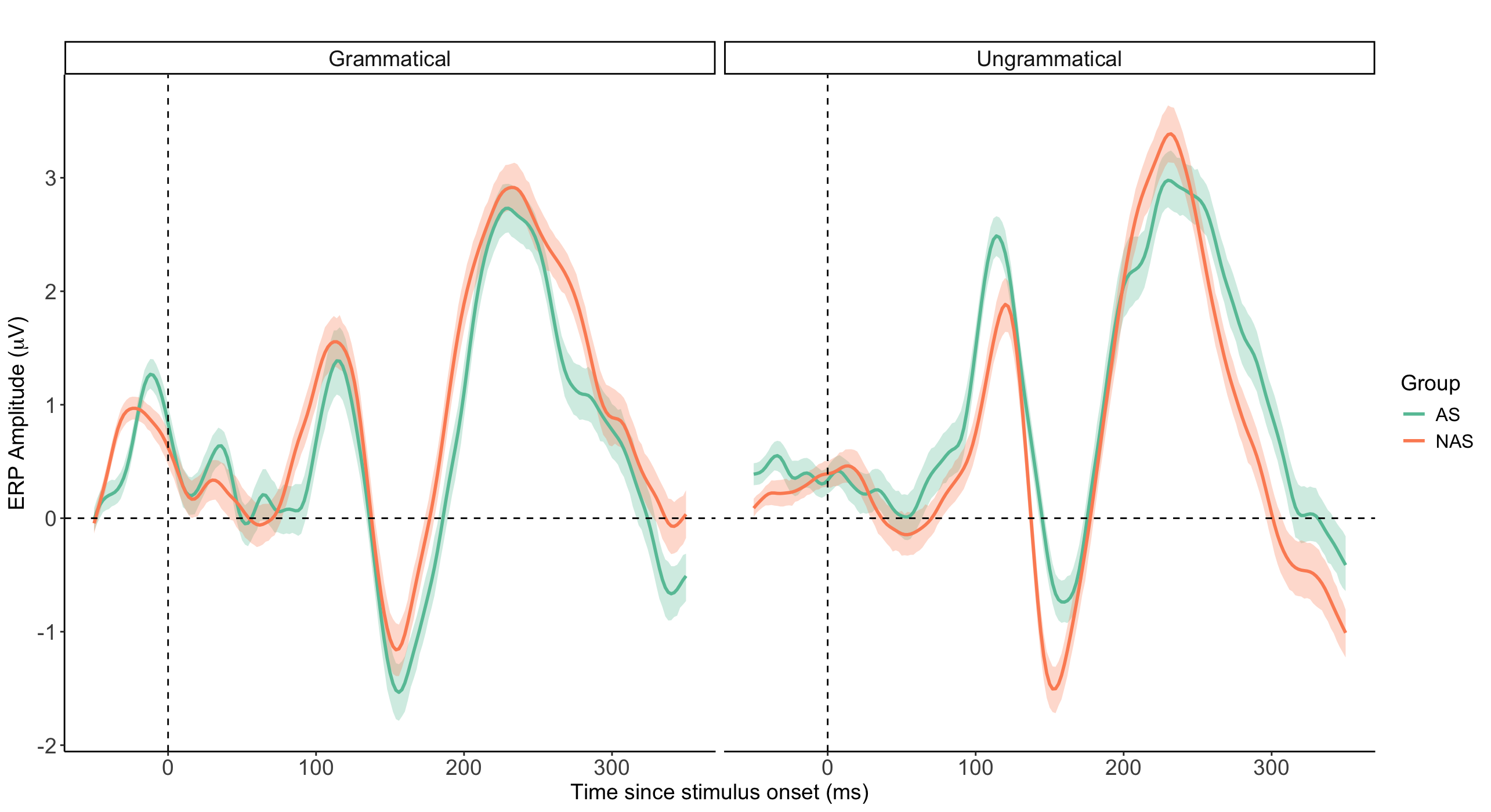


**3. ERAN & LAN analyses**

To provide a more comprehensive analysis of syntactic processing in language and music, we examined early ERP components associated with syntactic violations: the Early Right Anterior Negativity (ERAN) and Left Anterior Negativity (LAN). ERAN is a right-lateralized frontal and fronto-temporal negativity elicited by harmonic violations in music, whereas LAN is a left-lateralized anterior negativity in response to syntactic violations in language.

To assess both components across conditions, we defined two regions of interest (ROIs):

1) left anterior ROI: AF7, F1, F3, F5, F7, FC1, FC3, FC5, FT7;

2) right anterior ROI: AF8, F2, F4, F6, F8, FC2, FC4, FC6, FT8.

The time windows were set at 150–250 ms for ERAN and 300–450 ms for LAN, based on previous studies.

Separate two-way ANOVAs were conducted for ERAN in the music condition and LAN in the language condition. Each analysis included group (NAS vs. AS) as a between-subjects factor and grammaticality (grammatical vs. ungrammatical) as a within-subjects factor.

The results of these analyses, presented in Supplementary Table 3, revealed no significant main effects or interactions for ERAN or LAN amplitudes in either language or music conditions. This suggests that there was no significant ERAN or LAN responses to syntactic violations. ERP waveforms are provided in Supplementary Figures 4.

**Supplementary Table 3.** The results of ANOVAs examining differences in ERAN and LAN amplitudes between groups (autistic vs. non-autistic) and across conditions (grammatical vs. ungrammatical).

|  | ERP Components | **Factor** | | |
| --- | --- | --- | --- | --- |
| ROI |  | **Group** | **Condition** | **Group x Condition** |
| Left | ERAN | F(1, 60) = 0.97  *p* = 0.328 | F(1, 60) = 1.20  *p* = 0.279 | F(1, 60) = 0.49  *p* = 0.486 |
|  | LAN | F(1, 60) = 1.20  *p* = 0.279 | F(1, 60) = 1.19  *p* = 0.280 | F(1, 60) = 0.08  *p* = 0.782 |
|  |  |  |  |  |
| Right | ERAN | F(1, 60) = 0.13  *p* = 0.725 | F(1, 60) = 2.70  *p* = 0.106 | F(1, 60) = 2.04  *p* = 0.158 |
|  | LAN | F(1, 60) = 1.24  *p* = 0.269 | F(1, 60) = 0.42  *p* = 0.520 | F(1, 60) = 0.08  *p* = 0.778 |

**Supplementary Figure 4.** ERP waveforms for (a) the LAN responses (time window: 300-450ms) for language stimuli and (b) the ELAN responses (time window: 150-250ms) for music stimuli in both groups. The green lines depict responses to grammatical stimuli, while the orange lines represent responses to ungrammatical stimuli. Shaded areas indicate 95% confidence intervals. Electrode labels (e.g., AF7) are shown in the right-hand boxes. Time is on the x-axis, and ERP amplitude is on the y-axis.

**
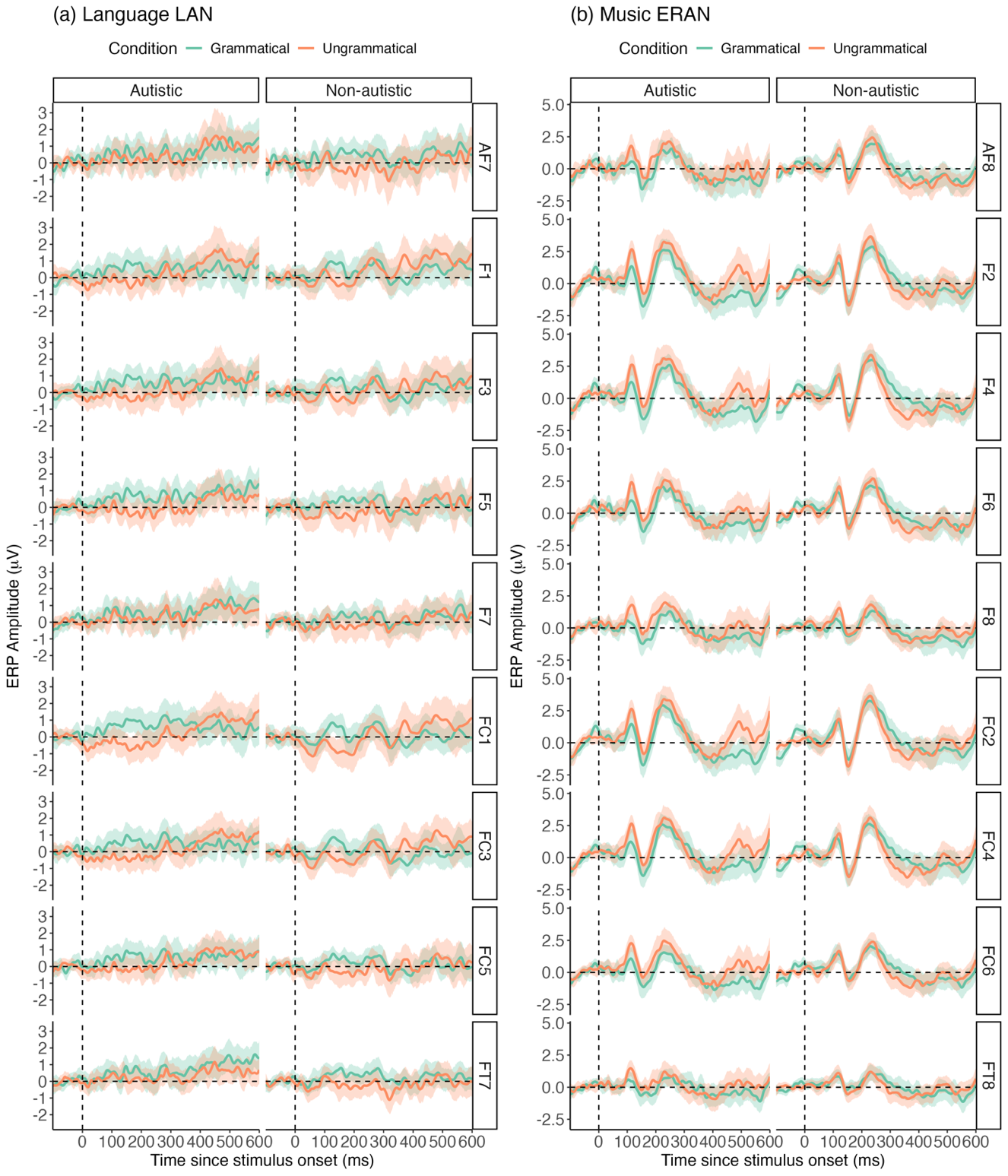
**

**4. Results for correlation analyses**

**Supplementary Figure 5.** Matrix for the (a) non-autistic and (b) autistic group displaying correlation coefficients for all variable pairs. More intense colours represent stronger correlations, while non-significant correlations (*p* < 0.05) are shown in white. Note: LangACC = behavioural accuracy in the language condition, MusicACC = behavioural accuracy in the music condition, LangP600 = P600 amplitude in the language condition, MusicP600 = P600 amplitude in the music condition, MBEA_T = total scores for Scale, Contour, and Interval subtests, MT = years of musical training.


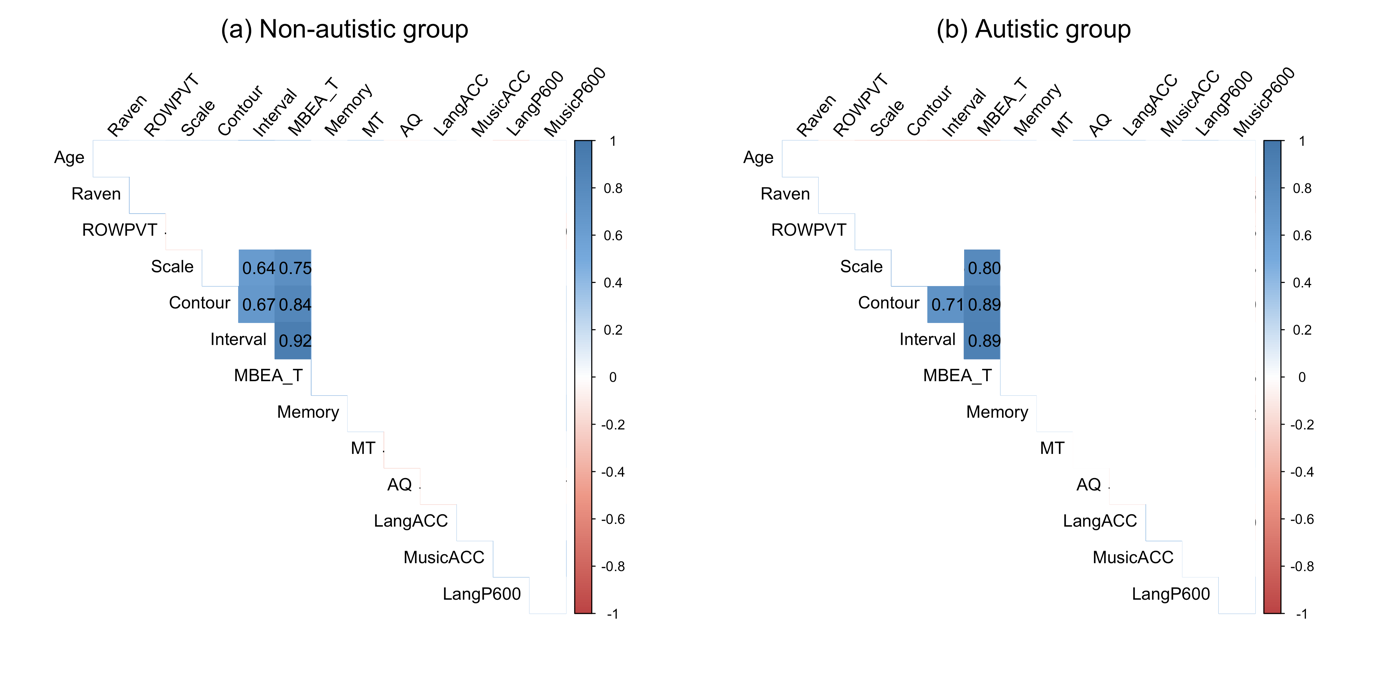


**References**

Hautus, M. J. (1995). Corrections for extreme proportions and their biasing effects on estimated values ofd′. *Behavior Research Methods, Instruments, & Computers*, *27*(1), 46–51. https://doi.org/10.3758/BF03203619

Makowski, D. (2018). The psycho Package: An Efficient and Publishing-Oriented Workflow for Psychological Science. *The Journal of Open Source Software*, *3*(22), 470. https://doi.org/10.21105/joss.00470

McClannahan, K. S., Backer, K. C., & Tremblay, K. L. (2019). Auditory evoked responses in older adults with normal hearing, untreated, and treated age-related hearing loss. *Ear and hearing*, *40*(5), 1106-1116. [https://doi.org/10.1097/aud.0000000000000698.](https://doi.org/10.1097/aud.0000000000000698. )
